# Supplementary material for: Antibacterial Properties of Melanoidins Produced from Various Combinations of Maillard Reaction against Pathogenic Bacteria
Source: Microbiol Spectr. 2021 Dec 15;9(3):e01142-21. doi: 10.1128/spectrum.01142-21 (PMC8672907; doi:10.1128/spectrum.01142-21)
Supplement: SUPPLEMENTAL FILE 1 — Supplemental material. Download SPECTRUM01142-21_Supp_1_seq4.pdf, PDF file, 0.1 MB [file spectrum01142-21_supp_1_seq4.pdf]

Table S1. Bacterial strains used in this study

Bacterial strains	Pathogenicity	Characteristics
**Gram-Positive**		
Bacillus cereus ATCC 10987	×	A non-pathogenic and aerobic strain isolated in 1930 by a study of cheese spoilage<br>Conducted in Canada<br>Xylose-positive variant
Brevibacillus brevis NBRC 100599	×	An aerobic and spore-forming bacterium<br>Isolated from soil in Japan<br>Consisting of a peptidoglycan layer and two outer S-layers<br>Produces a large amount of protein
Enterococcus faecalis ATCC 47077	×	Aerobic strain<br>A commensal inhabitant of the mammalian gastrointestinal tract<br>A human isolate subsequently shown to cause dental caries in rats<br>Resistance to rifampin and fusidic acid
Lactobacillus brevis JCM 1059	×	Isolated from human faeces<br>Aerobic strain (5% CO2 enhances growth)
Lactobacillus fructivorans NBRC 13954	×	Isolated from spoiled salad dressing in 1934<br>Aerobic strains<br>Synonym: *Lactobacillus heterohiochii*, and *Lactobacillus trichodes*
Listeria monocytogenes ATCC 19111	◯	Derived from poultry in England<br>Aerobic strain<br>Serotype 1/2a
Staphylococcus epidermidis ATCC 12228	×	Does not form a biofilm<br>Sensitive to vancomycin<br>Coagulase-negative<br>Unknown source of isolation
**Gram-negative**		
Escherichia coli ATCC 25922	×	Enterobacteriaceae<br>Aerobic strain<br>clinical isolate<br>Serotype: O6, Biotype 1
Escherichia coli O157: H7 HIPH 11361	◯	Foodborne disease strain
Salmonella Enteritidis RIMD 1933001	◯	Isolated from patients in a sporadic case<br>Serotype: O9: HG, m<br>invA gene +
Salmonella Typhimurium ATCC 29630	◯	Aerobic strain<br>Can be mated with Agrobacterium using a helper plasmid pRK2013 (ATCC 37159)<br>Independent replicon enables cloned insert to complement in trans<br>HisC3076, rfa-, uvrB derivative of strain LT-2
